# Amino acid nutrition of dairy cows: invited review

**DOI:** 10.5713/ab.260271

**Published:** 2026-05-07

**Authors:** Leoni F. Martins, Alexander N. Hristov

**Affiliations:** 1 Department of Animal Science, The Pennsylvania State University, University Park, PA, USA

**Keywords:** Amino Acids, Dairy Cattle, Histidine, Lysine, Methionine, Nitrogen Utilization

## Abstract

Improving nitrogen (N) efficiency in dairy production systems is important due to environmental concerns and the economic cost of dietary protein. A substantial proportion of dietary N consumed by lactating dairy cows is not converted into milk protein and is instead excreted in urine and consequently manure. Advances in protein nutrition have shifted feeding strategies from crude protein (CP)–based approaches toward metabolizable protein and, more recently, amino acid (AA)–based systems designed to better match absorbed AA supply with animal needs. This review synthesizes current knowledge on AA nutrition in lactating dairy cows, with emphasis on the biological regulation of milk protein synthesis, identification of limiting AA, and opportunities to improve N utilization efficiency through more precise diet formulation. Absorbed AA originate primarily from microbial protein synthesized in the rumen and rumen-undegraded feed protein digested postruminally, and the relative contribution of these sources influences both the quantity and profile of AA available for metabolism. Milk protein synthesis is regulated not only by AA supply but also by energy availability, endocrine signaling, and tissue nutrient partitioning. Among individual AA, methionine, lysine, and histidine are most consistently associated with limitations to milk protein synthesis in dairy cows under common feeding conditions. Evidence indicates that balancing diets for these AA can increase milk protein yield, improve feed N conversion into milk N, and allows reductions in dietary CP without compromising production. However, practical implementation of AA-based feeding strategies remains constrained by limitations in predicting microbial protein synthesis and postruminal AA supply. Nutritional models provide useful tools for evaluating AA adequacy, but prediction errors and biological variability require that model outputs be interpreted alongside production responses and metabolic context. Overall, improved alignment of AA supply with metabolic demand represents a key strategy for enhancing productivity while reducing environmental N losses in dairy production systems.

## INTRODUCTION

In lactating dairy cows, nitrogen use efficiency (NUE; defined as milk N, g/d÷feed N, g/d) typically ranges from 25% to 29% [1,2], although values between 13% and 46%, depending on stage of lactation, milk yield (MY), and N intake, have also been reported [3,4]. Milk N output may be reported with or without discounting non-protein N (NPN), which represents about 5%–6% of total milk N [5]. When considering the conversion of human-edible feed protein into human-edible animal protein, lactating dairy cows are more efficient than pigs and broiler chickens (130%–240% vs. 30%–50% and 70%–120%, respectively; [6]; reviewed by Hristov et al. [4]). This advantage stems from the unique ability of ruminants to convert high-fiber, human-inedible feeds into high-quality animal protein through their symbiotic relationship with rumen microorganisms. Nevertheless, concerns about the environmental impact of dairy production and the relatively low NUE of dairy cattle have intensified interest in nutritional strategies aimed at improving the conversion of feed N into milk protein while reducing excess N intake and excretion.

Historically, dairy diets were formulated primarily to meet targets for crude protein (CP), rumen degradable protein (RDP), rumen undegradable protein (RUP), and more recently metabolizable protein (MP). Although CP, RDP, RUP, and MP remain useful descriptors of dietary protein concentration, they do not adequately describe the quantity and profile of metabolizable amino acids (mAA) available to the animal. As a result, CP-based feeding strategies often lead to an oversupply of dietary N to ensure that mAA needs are met. Additionally, overfeeding CP increases feed costs without guaranteeing improvements in MY or milk components production. Protein is typically the most expensive nutrient in dairy diets. For example, the cost of soybeans is approximately 1.6 times that of corn grain on a USD/ton basis (based on 3-year average prices from USDA; [7,8]). Consequently, and depending on farm-gate milk price, excessive protein supplementation often results in diminished economic returns to the dairy farmer.

**Figure 1:**
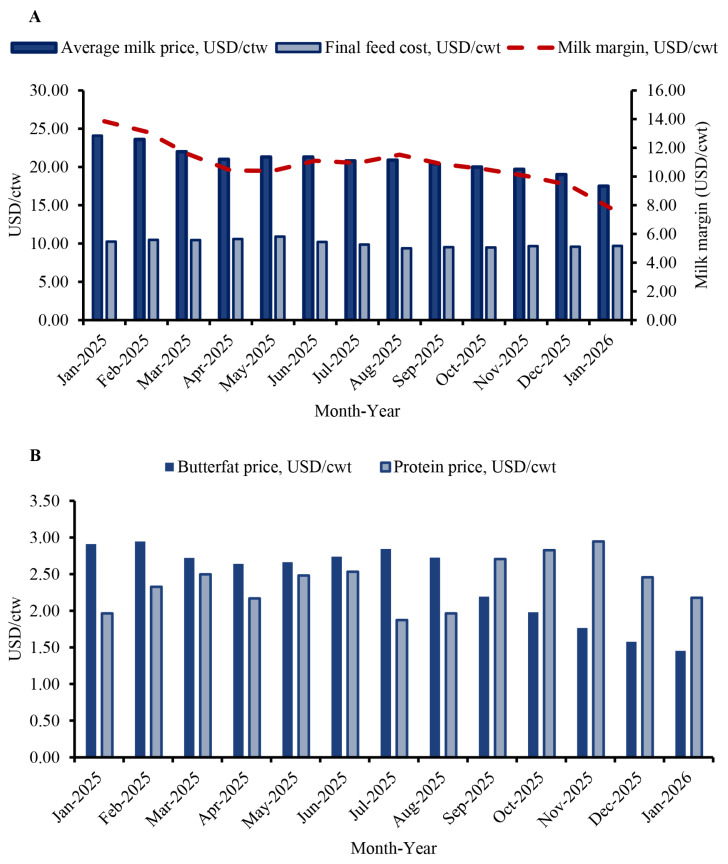
Average milk and feed prices paid to milk producers in the United States from January 2025 to 2026 [9]. (A) Average milk price (USD/cwt), final feed cost (USD/cwt) and milk margin over feed cost (USD/cwt) of dairy production; (B) Average butterfat and milk protein prices (USD/lb) paid to dairy producers.

The current milk margin scenario in the United States illustrates the importance of protein nutrition in dairy diets [9]. Milk margin over feed costs recently reached USD 7.81/cwt (i.e., hundredweight or 100 lb), a value 44% lower than the dairy margin observed in January 2025 (Figure 1A). Although milk component prices are often consistent with butterfat value exceeding milk protein value, current trends indicate that dairy farmers still have an opportunity to increase milk revenue by enhancing milk protein synthesis (Figure 1B)—a response that is typically more difficult to achieve nutritionally than increasing milk fat yield. Precision feeding can serve as a strategy to stimulate productive responses while improving N utilization [10,11]. Precision feeding approaches aim to improve ruminal and whole animal N balance so that dietary N is used more efficiently rather than being excreted in manure. Greater synchronization between energy and N supply in the rumen can enhance microbial protein synthesis, particularly through improved capture of recycled urea-N [12]. At the same time, balancing diets to deliver adequate mAA to the small intestine is considered a modern precision feeding strategy to maintain or enhance dairy cow performance while increasing milk protein yield.

However, important challenges remain. Accurate prediction of absorbed AA supply requires correct estimation of ruminal protein degradation, microbial protein synthesis, ruminal outflow of nitrogenous compounds, intestinal digestibility, and AA composition of both feed and microbial protein. Recent evaluations of nutritional systems demonstrate systematic biases in predicting AA outflow from the rumen and absorbed AA supply [13,14]. Moreover, productive responses to mAA supplementation are governed not only by substrate supply but also by metabolic regulation, endocrine signals, and nutrient partitioning among tissues [15,16]. Consequently, AA nutrition in dairy cows must be interpreted within the broader framework of whole-animal metabolism, and, therefore, mAA predictions from nutritional models should be interpreted with care.

The objective of this review is to synthesize current understanding of AA nutrition in lactating dairy cows with emphasis on (1) sources and metabolic fate of mAA, (2) physiological regulation of milk protein synthesis, (3) the role of methionine (Met), lysine (Lys), and histidine (His) as limiting AA, (4) limitations of current nutritional models in predicting AA supply, and (5) opportunities to improve N use efficiency through more precise AA balancing.

## THE ROLE OF RUMINAL NITROGEN METABOLISM

In the global context, and even in intensive production systems, microbial protein synthesis is the main source of mAA in dairy cows. In the review by Hristov et al. [4], the estimated proportion of microbial protein in total MP supply was 52% for cows producing on average 41 kg/d milk (based on NASEM [17] predictions). Broderick et al. [18] estimated the proportion of microbial N in the total non-ammonia N (NAN) outflow of about 68% in omasal flow studies. These averages are similar to the report by Martins et al. [14], in which the observed microbial N was 67.3% and 54.1% in omasal and duodenal outflow studies, respectively (Table 1). Martins et al. [14] also demonstrated variation in the predictions of microbial N outflow across nutritional systems (i.e., NRC [19]; Cornell Net Carbohydrate and Protein System [CNCPS], ver. 6.5.5; NASEM [17]; and NittanyCow, NittanyCow Software Services; https://nittanycow.com ), showing that there are limitations in the ability of these systems to predict ruminal N outflow. In that study and compared with observed values, CNCPS overpredicted NAN and microbial N outflows by 20.6% and 13.7%, respectively (p<0.001). Both NRC and NASEM overpredicted NAN by 14.0% and 11.9% and underpredicted microbial N by 8.1% and 6.5%, respectively. NittanyCow provided improved predictions for both NAN and microbial N compared with the other models [14].

Microbial growth depends on the interaction between fermentable energy supply and ruminal N availability. The source of ruminal N is also important for enhanced microbial protein production and overall efficiency. Most ruminal bacteria can grow with ammonia-N as their primary N source [20], although some microbial species benefit from peptides and preformed AA [21]. Studies using labeled N tracers and mixed or pure cultures consistently demonstrate that AA and peptides stimulate microbial growth, enhance microbial protein synthesis, and improve fiber digestion even when ammonia-N and fermentable carbohydrate are not limiting [22,23]. Similarly, Hristov et al. [24] reported an improved incorporation of ammonia-N into microbial N when α-amino-N was supplemented in a mixed-culture rumen medium containing NPN. However, energy efficiency for N utilization was lower when α-amino-N were used as the sole N source with a high-forage diet, compared with the combination of α-amino-N and ammonia-N [24], suggesting that cellulolytic bacteria used amino-N more efficiently in the presence of ammonia-N source. Hence, a combination of ammonia-N and α-amino-N, as well as supply of fermentable energy, are required for optimization of N utilization by rumen microbes. Recent evidence further supports and expands these observations. Räisänen et al. [25] evaluated the effects of supplementing individual free AA on ruminal fermentation and ^15^N enrichment of ruminal N pools *in vitro*. Their results showed that most AA produced only minor changes in overall fermentation end products or NDF digestibility; however, several AA noticeably modified fermentation pathways and ^15^N assimilation. Supplementation with branched-chain AA increased the corresponding branched-chain volatile fatty acids, and proline increased valerate concentration, confirming the activity of ruminal deamination and decarboxylation pathways. Notably, a group of AA (leucine, isoleucine, valine, methionine, tyrosine, tryptophan, histidine, phenilalanine, proline, glycine, cysteine, and arginine) significantly increased the incorporation of ammonia-N into microbial N without increasing total microbial biomass.

Rumen microbes flexibly adjust their reliance on ammonia versus amino-N depending on substrate supply and microbial community structure. Additionally, when ammonia-N is limiting, bacteria can retain and recycle N into intracellular pools [26], partially buffering short-term fluctuations in extracellular ammonia concentrations. Collectively, these findings indicate that rumen N requirements and microbial efficiency cannot be described solely by static ammonia-N targets. Rumen ammonia-N concentrations in the range of approximately 5 to 11 mmol/L generally appear sufficient to maximize microbial N flow, although no single optimal ammonia-N concentration can be recommended because microbial responses depend on diet composition, carbohydrate fermentability, and microbial community structure [27]. Additionally, microbial growth efficiency is constrained not only by substrate supply but also by intracellular energy utilization processes such as maintenance metabolism, reserve carbohydrate cycling, and energy spilling [27]. These processes reduce the proportion of fermented substrate recovered as microbial biomass and help explain why increasing RDP does not always translate efficiently into greater MP supply.

*In vivo* studies on the performance effects of NPN feeding in dairy cattle diets are not consistent. Broderick and Reynal [28] replaced solvent-extracted soybean meal with urea (increasing NPN from 20% to 44% of dietary N) and lignosulfonate-treated soybean meal in a diet with ~10.5% RDP and ~16% CP (DM basis). Increasing NPN linearly decreased microbial protein synthesis (using ^15^N as a marker) and its efficiency, indicating a negative effect of NPN on rumen fermentation. In contrast, Sannes et al. [29] reported no effect of protein source (urea vs. soybean meal) on microbial protein synthesis, dry matter intake (DMI), or production in cows fed diets with 17%–20% CP and 11%–13% RDP. Microbial protein synthesis, estimated from urinary purine derivatives, was 8% greater with soybean meal, although not statistically significant (likely due to methodological limitations and between-cow variation). The high CP in that study may have masked treatment effects due to elevated urea recycling and overall RDP supply. Similarly, Reynal and Broderick [30] showed that increasing RDP from 7.7% to 12.5% (DM basis; from feed RDP and NPN) linearly increased microbial protein outflow (omasal sampling; ^15^N) and microbial protein synthesis efficiency in early-lactation cows. Treatment did not affect DMI or MY, but milk true protein and rumen ammonia decreased with lower RDP. Overall, although ammonia-N can meet microbial N requirements, evidence suggests that microbial protein synthesis and rumen fermentation are enhanced by NAN sources, with potential benefits for production, particularly in high-producing cows.

## AMINO ACID SUPPLY TO THE LACTATING DAIRY COW

Over the past several decades, feeding systems have shifted toward the concept of MP, which represents the true protein absorbed in the small intestine after digestion [19]. More recently, advances in protein evaluation systems have emphasized prediction of digestible individual AA supply rather than total MP alone. Systems such as NASEM [17] and the CNCPS (ver. 6.5.5 [31]) attempt to characterize the metabolizable supply of essential AA and evaluate whether the profile of mAA are adequate to support maintenance, growth, reproduction, and lactation. Theoretically, this transition from MP based- to individual mAA systems should reflect the biological reality that milk protein synthesis is determined not only by total protein supply but also by the balance among mAA and their metabolic utilization by the cow as well as their interaction with metabolizable energy. Nevertheless, nutritional models are still limited in their ability to represent the whole biological system of a cow. For instance, immune function represents a major nutrient sink [32,33] but is not explicitly or implicitly considered in current requirement systems.

The term requirement suggests a biologically fixed value; however, AA needs vary considerably depending on physiological state, health status, production level, genotype, environmental conditions, nutrient interactions, and the response criterion of interest (e.g., maintenance, milk protein yield, fertility, or body tissue accretion). In addition, the complexity of ruminal metabolism limits our ability to precisely predict both the quantity and profile of AA that ultimately reach the small intestine for absorption. Consequently, the term requirement can be misleading, and alternative concepts such as recommended AA supply or adequate AA concentrations seem more appropriate. From a practical feeding perspective, protein and AA nutrition should therefore be approached as feeding for responses. In this framework, dietary protein and AA supply are adjusted to optimize economic return from animal products, most commonly MY and milk protein production, lean tissue deposition, and reproductive performance, while accounting for feed costs, management factors, and environmental constraints. This approach is context dependent and aims to identify the most economically efficient solution.

Among individual AA, Met, Lys, and His have received the greatest attention in dairy cattle nutrition. Methionine and Lys have long been considered the first-limiting AA in many soybean and corn-based diets [34,35], whereas His has emerged as particularly important in grass silage-based diets and in feeding scenarios where microbial protein constitutes the predominant source of mAA [36–39]. Numerous studies demonstrate that balancing diets more precisely for these AA can improve milk protein yield and NUE allowing reductions in dietary CP concentration [40,41].

The AA absorbed by lactating dairy cows originate primarily from microbial protein synthesized in the rumen and RUP digested postruminally. Endogenous secretions and recycled N also contribute indirectly to the total MP supply [17]. In many dairy diets, microbial protein constitutes the largest single source of absorbed AA, although the relative contribution of microbial versus dietary protein varies substantially with diet composition, intake level, and ruminal fermentation characteristics. The relative importance of RUP to the total MP supply increases when diets contain ingredients with lower ruminal protein degradability or when feed intake is high [42]. Because microbial protein and feed protein differ in AA composition and digestibility, the balance between these sources strongly influences the profile of AA reaching the small intestine.

These considerations form the basis of modern MP- and AA-based feeding systems. Under CP-based systems, diet formulation typically targets a specific dietary protein concentration. In contrast, MP-based systems attempt to provide sufficient RDP to support microbial growth while simultaneously supplying the host animal with adequate MP (microbial+ feed origins). More recent AA-based approaches further refine this concept by evaluating the supply of individual mAA relative to predicted needs [17]. Despite these advances, prediction of absorbed AA supply remains challenging. Uncertainties in estimating ruminal protein degradation, microbial protein synthesis, intestinal digestibility, or AA composition of microbial protein and feed ingredients can propagate into substantial errors in predicted mAA supply [43,44]. Therefore, although AA-based feeding represents a major advancement over CP-based approaches, effective application of these systems remains limited by the accuracy and precision of model predictions, requiring nutritionists to pay close attention to the response of cows when supplementing diets with individual rumen-protected AA (RPAA).

## PHYSIOLOGICAL REGULATION OF AMINO ACID UTILIZATION

There is evidence of physiological effects of AA in farm animals with examples among essential AA being leucine, which activates mammalian target of rapamycin (mTORC1) to stimulate protein synthesis and inhibit proteolysis, and tryptophan, which modulates neurological and immunological functions through multiple metabolites, including serotonin and melatonin [45]. According to Wu [45], certain AA, termed “functional”, can “*regulate key metabolic pathways to improve health, survival, growth, development, lactation, and reproduction of organisms*.” For a detailed discussion on physiological effects of AA, the reader is referred to the key review by Wu [45].

Milk protein synthesis depends not only on the supply of absorbed AA but also on the efficiency with which these mAA are utilized by mammary and extra-mammary tissues. After absorption, AA pass through the portal-drained viscera (PDV) and liver before entering systemic circulation, and a substantial portion may be metabolized by splanchnic tissues during this process, resulting in fractional losses before mAA become available to the mammary gland [46]. Modern dairy cows producing more than 45 kg/d of milk exhibit mammary blood flows exceeding 20 L/min [47] and total splanchnic flows approaching 50 L/min [48]. Considering the reported mammary flow:cardiac output ratio of 0.125 L/L from the study by Davis et al. [49], extrapolation to high-producing cows suggests cardiac outputs near 160 L/min, corresponding to circulatory turnover times of roughly 20 s. Under these conditions, essentially all circulating blood repeatedly passes through splanchnic tissues each minute, where mAA are subject to further removal.

As a result, post-absorptive inefficiency appears to be driven primarily by recycling losses rather than by first-pass extraction [46]. These recycling losses are amplified by incomplete extraction of mAA by target tissues. Fractional extraction by mammary tissue, liver, PDV, and peripheral tissues is well below 100% [50]. Because the circulatory system functions as a recycling network, a large proportion of mAA not extracted during each pass is returned to PDV and liver, increasing the cumulative probability of catabolism. This risk increases as circulating mAA concentrations rise with greater absorbed supply and decreases when blood mAA concentrations decline under reduced protein intake [46].

In addition, PDV, liver, and mammary tissues dynamically regulate mAA transport activity. In PDV and liver, clearance rate constants increase as arterial essential AA concentrations rise [46]. In contrast, mammary fractional extraction declines with increasing supply [51]. Mammary affinity is also influenced by energy supply [16,29] and by interactions among individual essential AA [50]. Overall, fractional extraction tends to decrease as circulating mAA concentrations increase, indicating that mammary uptake and utilization are saturable processes [51]. Consequently, increasing mAA supply does not necessarily produce proportional increases in milk protein synthesis. At the tissue level, specific substrates (e.g., acetate), hormones (e.g., insulin), and mAA (e.g., Met, Leu, Ile) further regulate protein synthesis and cell-signaling pathways [52–54].

Recent research has also emphasized the role of metabolic signaling pathways in regulating mammary protein synthesis. Pathways such as the mTORC1, the integrated stress response, and glycogen synthase kinase integrate signals from AA supply, glucose availability, and hormonal regulation to control rates of protein synthesis in mammary tissue [15]. Postabsorptive infusion studies demonstrate that glucose and essential AA act synergistically to stimulate mammary protein synthesis. Increasing glucose availability alone can enhance milk protein yield, partly through greater mammary mAA uptake and activation of anabolic signaling pathways [55]. For instance, glucose infusion increased both MY and milk protein yield and improved N utilization efficiency, partially by increasing mammary mAA clearance rates and stimulating anabolic signaling in mammary tissue [55]. In that study, simultaneous infusion of glucose and casein produced an additive increase in milk protein yield.

These findings were further supported by a meta-regression of 64 observations from 29 studies [56]. Increased glucose availability was associated with greater milk true protein yield, with an average glucose infusion of 954 g/d resulting in an increase of approximately 26 g/d of milk true protein synthesis. The relationship followed a second-order quadratic response to the infusion rate of glucose equivalents (i.e., glucose or propionate). Greater milk protein responses to glucose were observed when CP intake was maintained or increased, indicating that dietary strategies designed to increase glucogenic supply should likely be accompanied by adequate AA and protein supply. This is a finding also observed through the relationship between MY and milk protein yield (see below). When CP intake declined during glucose-equivalent infusion, the stimulatory effect of glucose on milk true protein yield was reduced [56]. Collectively, these observations indicate that milk protein synthesis is regulated by the interaction among mAA supply, energy availability, endocrine signals, and tissue-level nutrient partitioning. Consequently, productive responses to RPAA supplementation often depend strongly on physiological state, energy balance, and overall nutrient supply.

## INCORPORATION OF AMINO ACIDS IN DAIRY COW NUTRITION

Recent advances in AA nutrition have highlighted the potential of Met, Lys, and His to enhance lactational performance and improve dairy cow responses to dietary supplementation. However, when fine-tuning dietary strategies to maximize performance, it is essential to view the dairy cow as an integrated biological system in which diet composition, feeding management, rumen function, and postabsorptive metabolism interact. In practice, nutritional strategies that increase milk protein yield frequently also increase total milk production, reflecting the close biological linkage between milk protein and lactose synthesis. This relationship arises because α-lactalbumin functions as the capacity-determining subunit of lactose synthetase [57]. Consequently, increased synthesis of *α*-lactalbumin recruits additional galactosyltransferase into the lactose synthetase complex, increasing enzymatic capacity and stimulating protein production.

**Figure 2:**
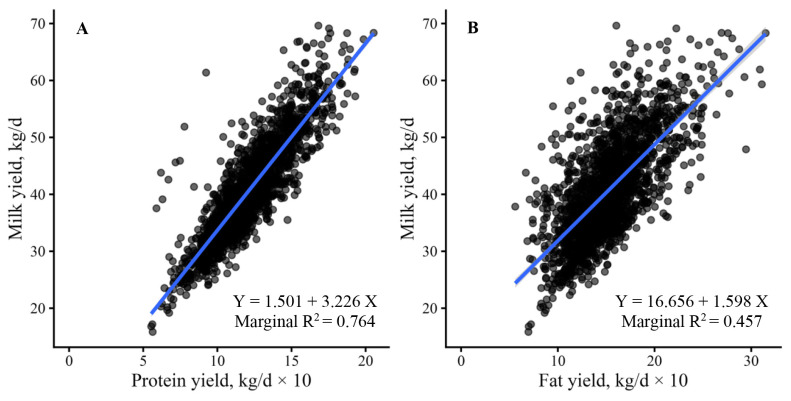
Relationship among milk protein yield (kg/d×10; A) and milk fat yield (kg/d×10; B) and milk yield (kg/d) in Holstein dairy cows (n = 1,976 and 1,987 observations for A and B, respectively) from 46 studies conducted at The Pennsylvania State University (Data from Hristov et al. [4]). Marginal R^2^ measures the proportion of variance explained by the fixed effects, excluding random effects. Data were analyzed using linear mixed effects models in R (ver. 4.5.1). Statistical models were fitted with the fixed effect of milk protein yield (A) or milk fat yield (B) and the random effect of study.

Evidence from a large dataset of individual dairy cows (n = 1,987 observations) enrolled in 46 studies at The Pennsylvania State University illustrates this relationship. A 0.1 kg increase in milk true protein yield was associated with 3.23 kg increase in MY (p<0.001; Figure 2A). The marginal R^2^ was 0.76, indicating that 76% of the variation in MY response was explained by changes in milk true protein yield, and the conditional R^2^ was 0.84 when accounting for both fixed and random effects (i.e., random effect of study). The relationship between milk and milk fat yield is less prominent. A 0.1 kg increase in milk fat yield was associated with 1.60 kg increase in MY (p<0.001; Figure 2B), with marginal and conditional R^2^ of 0.46 and 0.59, respectively. The intercepts of both equations also help illustrate the stronger association between MY and milk protein yield than milk fat. In a hypothetical scenario where milk protein or milk fat yield is 0, cows would be expected to produce 1.50 or 16.7 kg/d MY, respectively. As previously described, these relationships can be partly explained by increased glucose supply and insulin-like anabolic responses [15]. These responses differ from non-insulin-like mechanisms (e.g., acetate or fatty acid supplementation; [15]), which are more closely associated with stimulation of milk fat synthesis. Diets that increase ruminal propionate production (such as those with greater starch concentration) also commonly enhance microbial protein synthesis, which provides a more balanced AA profile than most feed proteins. Overall, increases in MY are typically accompanied by increases in milk true protein yield, and vice versa. This concept is important because it frames AA nutrition as a strategy to improve whole-lactation productivity and efficiency, rather than simply altering milk composition.

## RESPONSES TO AMINO ACIDS SUPPLEMENTATION IN DAIRY COWS

In practical dairy nutrition, milk protein synthesis is often limited not only by total MP supply but by imbalances among absorbed AA or inadequate energy supply. When one essential AA is deficient relative to metabolic demand, the remaining AA cannot be used efficiently for protein synthesis and are instead deaminated, increasing urinary N excretion and reducing the efficiency of MP utilization. Evidence from swine and poultry research suggests that optimal AA profiles likely exist for different physiological functions. If such profiles could be defined for cattle—and if both the total supply and balance of absorbed AA could be optimized—it is still uncertain how much the efficiency of MP utilization for maintenance and productive functions would improve.

Even under theoretically ideal AA nutrition, considerable and unavoidable mAA losses would persist because of the dynamic and inherently inefficient nature of normal physiological processes [58]. Ultimately, all AA can be oxidized to CO_2_ and H_2_O and therefore serve as direct sources of metabolic energy. Beyond their role in protein synthesis, AA also participate in numerous metabolic pathways. For example, all AA except Lys and Leu can act as precursors for gluconeogenesis, and all AA can be converted into fatty acids [17]. The identity of the first-limiting AA depends strongly on diet composition, production level, and the relative contributions of microbial protein and RUP to total MP supply. These factors highlight the importance of balancing diets for appropriate proportions of individual AA rather than relying solely on total dietary CP concentration or predicted MP supply.

### Methionine

Methionine is among the most extensively studied AA in lactating dairy cows and consistently emerges as one of the most important limiting AA in practical feeding systems. Numerous studies show that supplementation with rumen-protected Met or postruminal Met infusion increases milk protein yield and often increases milk fat yield and total milk production [59–61]. Responses to Met supplementation (and to increased MP supply in general) tend to be particularly pronounced during early lactation, when cows experience high metabolic demand and the marginal efficiency of nutrient use is elevated.

During the transition period, postpartum Met supplementation has been reported to increase DMI, MY, and milk fat and milk protein yields [60]. In their meta-analysis of 21 studies including 40 treatment–control comparisons, four Met sources were evaluated: 2-hydroxy-4-(methylthio)butanoate (HMTBa; methionine analog) and 3 commercial RPAA sources, with assumed bioavailability (BA) coefficients of 40 and 50%–80% of Met intake, respectively. Prepartum supplementation of rumen-protected Met (8.20±2.94 g/d mMet) did not affect DMI, body weight (BW), or body condition score (BCS), whereas postpartum supplementation (10.53±3.30 g/d mMet) increased lactational performance, including DMI, MY, and milk component yields. The duration of supplementation averaged (mean±SD) 19.3±4.23 d prepartum and 85.9±38.36 d postpartum. At 21 days in milk (DIM), responses were 1.38±0.283 kg/d DMI, 2.13±0.515 kg/d MY, 118±23 g/d milk fat, and 92±18 g/d milk protein greater for mMet vs. control cows. These responses tended to decline as lactation progressed, suggesting that physiological state strongly influences responsiveness to mMet supplementation. Beyond its role as a substrate for protein synthesis, Met also participates in one-carbon metabolism, methylation reactions, and antioxidant pathways. These broader metabolic roles may contribute to improvements in metabolic health and immune status reported in some transition cow studies [62,63].

Postpartum cows experience negative energy balance [33] and negative MP balance [64] and may lose approximately 30%–35% of longissimus dorsi muscle reserves [65]. This negative energy and AA balance likely reflects both reduced DMI after calving and increased energy and AA demand associated with immune activation and inflammation [33,66]. Consistent with this concept, greater MP supplementation during the first 3 weeks of lactation has been shown to improve MY and milk protein yield [67,68]. However, adequate energy supply must accompany increased protein supplementation to support these productive responses and avoid exacerbating body fat mobilization [69]. It remains unclear whether optimizing the profile of MP, rather than increasing total MP supply, could further improve transition cow performance or what the optimal MP profile should be.

In contrast with meta-analyses evaluating Met supplementation in established lactation [59], responses during the transition period were not dose-dependent and tended to decline as lactation progressed. In the analysis of Zanton and Toledo [60], the DIM at the end of supplementation influenced response magnitude, with supplementation earlier in lactation producing greater responses than supplementation later in lactation. Methionine supplementation increased DMI, MY, and milk component yields through both greater milk production and higher milk fat and true protein concentrations. When responses were evaluated by source, HMTBa was the only source that did not increase milk true protein concentration. These results were consistent with the earlier meta-analysis by Zanton et al. [59] conducted in established lactation. In that report, responses ranged from −0.25 to 0.31 kg/d DMI, −0.34 to 0.31 kg/d MY, 6 to 45 g/d milk fat yield, and 13 to 35 g/d milk protein yield depending on mMet supplementation rate. Overall, Zanton et al. [59] estimated a 2.23 g/d increase in milk protein yield for each additional g of mMet supplemented, up to a breakpoint of approximately 65 g/d of supplemental mMet. It is noted, however, that this breakpoint may be greater in modern cows producing more than 36 kg/d MY—the average milk production of cows in the meta-analysis by Zanton and Toledo [60].

In the meta-analysis by Zanton et al. [59], the regression response in milk fat yield was 1.87 g fat/g mMet for infused Met and RPMet whereas the response to HMTBa was significantly greater at 5.38 g fat/g mMet. These results suggest that Met sources may be equivalent in terms of mMet-driven milk protein responses but differ in associated milk fat responses, potentially reflecting additional ruminal effects of HMTBa beyond its role as a Met source. A broader database compiled by Feng et al. [70] reported that HMTBa supplementation increased milk fat yield by approximately 45 g/d and milk protein yield by 13 g/d, while emphasizing that responses in MY and milk protein yield were more variable across studies than responses in milk fat yield.

Collectively, Met supplementation produces a consistent milk protein yield response with diminishing production returns, although the magnitude and nature of responses depend on Met source, basal diet composition, and the presence of other limiting nutrients. Subsequent meta-analyses have further confirmed that Met supplementation increases energy-corrected milk, milk protein yield, and milk fat yield, although response magnitude is influenced by parity, stage of lactation, basal diet composition, MP supply, and supplementation duration [41].

### Lysine

Lysine is commonly considered co-limiting AA with Met in corn-based dairy diets [34]. Supplementation with rumen-protected Lys has been associated with increases in MY, milk protein yield, and milk protein concentration [71,72]. Compared with Met, responses to Lys supplementation are often smaller but remain biologically meaningful, particularly in Lys deficient diets (i.e., corn grain and corn protein supplement-based diets). In many cases, responses to Lys depend on adequate Met supply, highlighting the importance of balancing AA profiles rather than correcting single deficiencies in isolation. Increasing the proportion of digestible Lys within MP has been associated with improvements in milk production and feed efficiency, particularly during early lactation and in diets where predicted Lys supply is marginal relative to needs [71].

Recent hierarchical meta-analyses indicate that Lys supplementation increases MY (weighted mean difference [WMD] = 0.52 kg/d relative to control), milk protein yield (WMD = 0.03 kg/d), and milk protein concentration (WMD = 0.05%), with particularly strong responses observed in early lactation and in cows fed corn silage–based diets [72]. Milk fat yield also increased with Lys supplementation (WMD = 0.02 kg/d relative to control). However, these responses were generally smaller than those reported for Met supplementation. Importantly, Lys responses were often greater when Met supply was adequate, demonstrating a clear interaction between these two AA. In addition, Lys supplementation appeared more effective in MP-adequate diets, contrasting with findings for Met supplementation, where responses in MY, milk fat yield, and milk protein yield were often more pronounced under MP-deficient conditions [41].

In a meta-analysis of 13 experiments (40 treatment means), Arshad et al. [71] reported that cows supplemented with RPLys received an additional 19.3±10.3 g/d mLys (range 5.1 to 40.6 g/d). Productive responses depended on both the timing and duration of supplementation. Milk yield increased by 1.51 kg/d compared with controls when supplementation began in early lactation (≤90 DIM) or lasted at least 70 d. Increasing digestible Lys in MP from 6.5% to 8.5% resulted in linear increases of 1.8 kg/d MY, 2.5 kg/d fat-corrected milk, 2.4 kg/d energy-corrected milk, and 0.10 kg/d milk fat yield, with a tendency for milk protein yield to increase by 0.07 kg/d. These responses occurred without a concurrent increase in DMI. Arshad et al. [71] also reported interactions between Lys and Met concentrations in total MP affecting feed efficiency, such that increasing Met strengthened the positive response to greater Lys concentration in MP. Unlike Met, Lys responses appear to depend more strongly on the relative proportion of Lys within MP rather than absolute intake alone. Increasing the proportion of digestible Lys in MP may be associated with improvements in milk production and feed efficiency, particularly when Met supply is adequate. Changes in circulating AA profiles following Lys supplementation further suggest both competitive and cooperative interactions among AA pools, indicating that increased Lys supply can alter AA partitioning between mammary and extra-mammary tissues.

### Histidine

Histidine has emerged as an important limiting AA in several dairy feeding situations. Early studies showed that His supplementation increased milk protein yield in cows fed grass silage–based diets, whereas supplementation with Met and Lys alone did not produce similar responses [36,37]. One explanation for this response relates to the AA composition of microbial protein. Histidine represents a smaller proportion of microbial protein relative to its concentration in milk protein, whereas Lys and Met concentrations are more similar between these protein sources. As a result, diets in which microbial protein constitutes a large proportion of MP may predispose cows to His limitation [73].

More recent evidence supports His as a limiting AA under specific dietary conditions, especially low-CP or MP-deficient rations with high reliance on microbial protein. Räisänen et al. [39] summarized data from 17 publications (22 studies) and reported that His supplementation increased plasma His concentration (standardized mean difference [SMD] = 1.39 mM), DMI (SMD = 0.24 kg/d), MY (SMD = 0.68), milk true protein concentration (SMD = 0.24%), and milk true protein yield (SMD = 0.58 g/d). Similar findings were reported in a subsequent meta-analysis by Xu et al. [74]. In that analysis, His supplementation increased DMI, MY, and milk true protein yield, with responses often larger than those reported for Met or Lys under comparable MP-deficient conditions. These responses were particularly pronounced in cows fed grass silage– or corn silage–based diets with high reliance on microbial protein, reflecting the relatively low His content of microbial AA. Importantly, responses to His supplementation were strongly influenced by MP supply, basal diet composition, and supplementation method [39,74]. Xu et al. [74] further suggested that total mHis supply of approximately 72.6 g/d maximizes milk protein yield in dairy cows. Increases in milk true protein concentration and yield were reported to be 3.9× and 1.3× greater, respectively, in MP-deficient compared with MP-adequate diets [39] according to NRC [19] classification.

A distinctive aspect of His metabolism is the presence of sizable endogenous labile pools, including hemoglobin and muscle dipeptides, which can temporarily buffer His deficiency. These pools may mask short-term deficiencies and contribute to delayed or nonlinear responses to supplementation. However, once these reserves are depleted, His can become acutely limiting for milk protein synthesis [42]. Meta-analyses also indicate that the optimal digestible His supply for maximizing milk protein yield is finite and nonlinear, suggesting that the efficiency of His utilization declines once supply exceeds metabolic demand [39]. Their regression analyses further revealed nonlinear responses of DMI, MY, and milk true protein yield to increasing mHis supply, and response magnitude differed by supplementation method, with larger effects often observed when His was infused compared with rumen-protected supplementation. In addition, an energy interaction was identified, whereby the efficiency of His utilization declined as the ratio of digestible His to net energy for lacatation (NE_L_) [39] increased, highlighting that AA utilization is constrained by the broader energetic and metabolic context.

## BIOAVAILABILITY OF RUMEN-PROTECTED AMINO ACIDS

Accurate estimation of BA of RPAA remains one of the main limitations to the effective implementation of AA–based feeding systems in dairy cattle. Bioavailability is defined as the proportion of an ingested AA that escapes ruminal degradation, is digested postruminally, and becomes available for metabolism. Consequently, BA is a function of both rumen escape (RE) and intestinal digestibility, and errors in either component directly affect estimates of mAA supply.

Räisänen et al. [75] evaluated BA of multiple RPAA products using a method based on RE (*in situ*) combined with fecal excretion of undigested AA. Bioavailability was calculated as the product of RE and post-ruminal digestibility. The study demonstrated substantial variability among products, with BA ranging from approximately 58% to 76% for His, 10% to 67% for Lys, and 49% to 92% for Met. This wide range highlights the strong influence of protection technology and product characteristics on both ruminal stability and intestinal release. Importantly, the study showed that even when products are designed to supply the same digestible AA, their effective contribution can differ markedly. Differences in BA were largely driven by variation in RE and intestinal digestibility, both of which are affected by coating material (e.g., lipid vs. polymer), particle size, and physicochemical properties of the product. In addition, BA based on manufacturer data may be different from experimentally derived BA for some RPAA.

A subsequent study by Räisänen et al. [76] directly compared multiple *in vivo* methodologies for estimating BA, including plasma-based approaches [area under the curve (AUC) and plasma dose-response (PDR)] and the fecal free AA (FFAA) method. Estimates of BA differed substantially across methods. The AUC method yielded lower, relative BA values (e.g., ~45% for His, ~72% for Lys, and 43% to 61% for Met), whereas the FFAA method produced higher estimates (e.g., ~87% for His, ~75% for Lys, and 67% to 91% for Met). Despite these differences in magnitude, both methods ranked products similarly, indicating that relative comparisons among products are consistent even when absolute values differ. The PDR method was highly sensitive to experimental conditions and produced unrealistic estimates in that study, emphasizing the importance of strict methodological control when using plasma-based approaches.

These studies demonstrate that BA estimates are method-dependent. Plasma-based methods (e.g., AUC, PDR) quantify AA appearance in circulation and therefore provide relative estimates of BA, which are influenced by absorption kinetics, sampling frequency, and metabolic utilization of AA. In contrast, fecal-based approaches incorporate both RE and intestinal digestion, providing estimates closer to true digestible AA supply. However, each method has limitations. Plasma methods may underestimate BA due to splanchnic metabolism of absorbed AA, whereas fecal methods rely on accurate estimation of RE and complete recovery of undigested AA in feces. In addition, differences in experimental design (e.g., dosing strategy, feeding frequency) can substantially influence outcomes, particularly for plasma-based techniques.

The variability in BA across products and methodologies has important implications for diet formulation. Use of inaccurate or non-standardized BA values can lead to over- or under-supply of mAA, contributing to inconsistent production responses and reduced N use efficiency. The data also reinforce that RPAA products cannot be treated as nutritionally equivalent, even when labeled for the same mAA. Overall, current evidence supports the need for standardization of *in vivo* methodologies and more robust characterization of RPAA products under practical feeding conditions. Until such standardization is achieved, caution is warranted when applying BA values across studies or relying solely on manufacturer specifications.

## LIMITATIONS OF NUTRITIONAL MODELS PREDICTING AMINO ACID SUPPLY

Modern protein evaluation systems represent a major improvement over earlier CP-based approaches, yet substantial uncertainty remains in predicting AA supply to the small intestine. Comparative evaluations of NRC [19], NASEM [17], and CNCPS [31] have identified both mean and linear biases in predicted AA flows [13]. Linear bias is particularly problematic because prediction error changes with AA supply, potentially altering the ranking of diets across feeding conditions. Among individual AA, His appears especially difficult to predict, which is notable given increasing evidence that His can be limiting in several common feeding scenarios [13].

Part of this apparent model error reflects inconsistencies in the experimental reference data used for evaluation. Measurements of microbial protein and AA flows differ depending on sampling site (omasal vs. duodenal) and the microbial markers used to estimate microbial protein flow [13,43]. Omasal sampling generally yields greater estimates of microbial N and AA flow than duodenal sampling, and marker choice (e.g., purines vs. ^15^N) further affects observed values. Consequently, the apparent accuracy of model predictions depends strongly on the dataset used for comparison.

Uncertainty in microbial protein prediction represents a central limitation in estimating AA outflow. Current systems rely on empirical relationships linking fermentable energy supply to microbial protein synthesis, yet microbial N outflow shows substantial unexplained variation across studies [14,43]. Because microbial protein contributes a large proportion of absorbed AA and is assumed to have a relatively consistent AA profile, errors in microbial flow prediction directly translate into biased estimates of individual AA supply.

Additional uncertainty arises from reliance on feed library values for protein fractions and AA composition. When measured feed ingredient nutrient composition is incorporated into model evaluations, prediction bias in AA outflow is reduced, indicating that part of the apparent model error originates from uncertainty in feed composition inputs [13]. Furthermore, many datasets used to develop ruminal outflow equations represent earlier production conditions with lower feed intake and MY than modern dairy systems. Extrapolation to contemporary high-producing cows therefore increases the likelihood of systematic prediction bias [14].

Taken together, these limitations indicate that current nutritional models, while useful for general ration formulation, remain imperfect tools for predicting individual AA supply. Within this context, response-based and absorbed-AA approaches provide a practical alternative for guiding Met, Lys, and His nutrition. Because predictions of postruminal AA supply (particularly His) are subject to systematic uncertainty, models that relate milk protein yield directly to absorbed essential AA supply and available energy may offer a more robust framework for identifying co-limitation and prioritizing supplementation strategies [16]. Evidence indicates that variation in milk protein synthesis is more consistently associated with estimated absorbed essential AA supply than with predicted ruminal AA flows, reflecting the closer physiological link between absorbed AA and mammary protein synthesis.

## PRACTICAL EVALUATION OF AMINO ACID NUTRITION USING NUTRITIONAL SYSTEMS

Modern ration formulation models such as NASEM [17] and CNCPS [31] estimate mAA supply and help identify potential AA limitations in dairy diets. Both systems are based on the principle that mAA originate primarily from microbial protein synthesized in the rumen and RUP digested postruminally. Microbial protein generally supplies the majority of MP in dairy diets, although the contribution of RUP increases as less degradable protein supplements are included in the diet. Predicted AA supply is then evaluated relative to estimated needs for maintenance, growth, reproduction, and milk protein synthesis.

In the NASEM [17] framework, MP is partitioned into digestible individual essential and non-essential AA, and AA needs are estimated using factorial approaches based on tissue composition and efficiencies of AA utilization. For lactating cows, the adequacy of AA supply has traditionally been assessed using the predicted proportions of mLys and mMet in MP, with commonly suggested targets of approximately 6.5% to 7.2% Lys and 2.2% to 2.4% Met. However, these targets should be interpreted cautiously because the true limiting AA profile depends on diet composition, production level, and the relative contributions of microbial protein and RUP. The CNCPS [31] model similarly predicts ruminal protein degradation, microbial protein synthesis, and postruminal AA supply based on feed fractionation and ruminal fermentation characteristics, estimating flows of individual AA to the small intestine and evaluating potential limitations relative to predicted requirements.

Despite these advances, model predictions should be interpreted as decision-support tools rather than precise measurements of AA supply. In practice, nutritionists often combine model outputs with empirical indicators such as milk urea N concentration, milk protein yield, feed efficiency, and production responses to dietary adjustments. Effective implementation of AA-based feeding strategies, therefore, requires integrating model predictions with biological understanding of rumen fermentation, energy metabolism, and lactation physiology. Both models also provide reference efficiencies for AA utilization that can help identify potential imbalances in dietary supply (Table 2). When predicted AA efficiency exceeds the model target, supply may be insufficient relative to demand, whereas efficiencies lower than the target suggest potential oversupply. Although fixed efficiencies are not biologically exact—because AA utilization varies with physiological state, AA supply, energy supply, and other dietary factors—ranking AA efficiencies within a model can still provide useful insight into relative limitations.

To illustrate how model predictions may differ, practical formulation scenarios were evaluated using both NASEM [17] and CNCPS [31]. Diets were formulated for a multiparous Holstein cow producing 45 kg/d MY with 4.2% milk fat and 3.4% milk true protein, BW of 650 kg, BCS of 3.0, 150 days-in-milk, and DMI of 28 kg/d. For comparability, the same feed ingredients were used in both systems and only CP, NDF, ADF, and starch concentrations were adjusted; other nutrient values remained at default library values. Diets for CNCPS [31] simulations were formulated using NDS (Nutrition Dynamic System; RUM&N). Two scenarios were evaluated. In the basal scenario, only basal ingredients were included, allowing comparison of model predictions without supplemental RPAA. In the second scenario, rumen-protected Met (11 g/d mMet), Lys (15 g/d; mLys), and His (11 g/d; mHis) were added to balance AA supply according to NASEM [17] target efficiencies (Table 3).

Under the basal diet, NASEM [17] predicted greater energy-allowable milk (47.6 kg/d) but substantially lower MP-allowable milk (36.5 kg/d) than CNCPS [31] (47.0 and 45.0 kg/d, respectively). As a result, predicted milk true protein yield was lower in NASEM [17] (1.29 kg/d) than in CNCPS [31] (1.42 kg/d). Supplementation with RPAA increased predicted milk protein yield in both models, although the magnitude differed: NASEM [17] predicted an increase from 1.29 to 1.34 kg/d, whereas CNCPS [31] predicted a smaller increase from 1.42 to 1.44 kg/d. Dietary nutrient composition was generally similar between models, although CNCPS [31] predicted slightly lower NDF and ADF concentrations. A major contrast occurred in predicted MP balance: NASEM [17] indicated a substantial MP deficit (−422 g/d in the basal diet, improving to −367 g/d with RPAA supplementation), whereas CNCPS [31] predicted near adequacy under the basal diet (−0.4 g/d) and a small surplus (+32.4 g/d) with RPAA supplementation. The CNCPS [31] also predicted consistently greater supplies of several essential AA (e.g., Lys, Met, His) than NASEM [17], even though both models predicted similar relative increases when supplemental AA were included.

Overall, these simulations indicate that the two systems produce similar predictions for energy supply and diet composition but diverge considerably in predicted MP supply and AA availability, which ultimately influences predicted milk protein responses. Differences in the prediction of NAN, microbial protein synthesis and individual AA outflow appear to contribute to these discrepancies: in the basal diet, predicted concentrations of Met, Lys, and His were approximately 27%, 15%, and 45% greater in CNCPS [31] than in NASEM [17], respectively.

## CONCLUSION

Amino acid nutrition is central to modern protein feeding strategies for lactating dairy cows. Although CP and MP remain useful organizing concepts, the biological control of milk protein synthesis is increasingly understood in terms of the amount, balance, and metabolic utilization of absorbed AA. Among individual AA, Met, Lys, and His frequently play key limiting roles in dairy cow production, but their responses depend strongly on diet composition, stage of lactation, energy supply, and whole-body nutrient partitioning. Evidence indicates that balancing diets for limiting AA can improve milk protein yield and NUE while reducing reliance on high-CP diets. However, the practical application of AA-based feeding remains constrained by uncertainty in predicting AA supply, particularly ruminal protein degradability, microbial protein synthesis, and individual AA flows to the small intestine. As a result, model outputs should be interpreted cautiously and integrated with biological knowledge of rumen fermentation, energy metabolism, and lactation physiology. Overall, AA nutrition in dairy cows should be viewed as an integrated metabolic problem rather than a simple first-limiting nutrient exercise. Future progress will depend on improved characterization of feed AA composition, more accurate prediction of microbial and feed postruminal AA supply, and nutritional frameworks that better integrate AA nutrition with energy metabolism and physiological state of the animal.

## Figures and Tables

**Table 1:** Mean and standard deviation (SD) of microbial N proportion relative to the total non-ammonia N ruminal outflow in 252 studies published in the literature and reviewed by Martins et al. [14]

Model	Sampling site	Mean	SD	n
Observed	Duodenal	54.1	11.15	185
	Omasal	67.3	7.36	67
NRC [19]	Duodenal	54.0	5.96	185
	Omasal	56.4	5.79	67
CNCPS (ver. 6.5) [31]	Duodenal	62.1	6.53	185
	Omasal	64.7	5.82	67
NASEM [17]	Duodenal	56.0	7.06	185
	Omasal	58.7	4.94	67
NittanyCow	Duodenal	56.2	5.94	185
	Omasal	60.1	5.61	67

NRC, National Research Council; CNCPS, Cornell Net Carbohydrate and Protein System; NASEM, National Academies of Sciences, Engineering, and Medicine.

**Table 2:** Target or estimated AA efficiencies for NASEM [17] and CNCPS models, and overall recommendations for AA supplementation relative to metabolizable energy intake and total essential AA according to CNCPS

Amino acids	Target or estimated AA efficiencies			CNCPS (ver. 7)^2)^	

	NASEM [17]	CNCPS (ver. 6.5) [31]^1)^	CNCPS (ver. 7)^2)^	g AA/Mcal ME^3)^	% EAA^4)^
Arg	-	58	61	2.04	10.2
His	75	76	77	0.91	4.5
Ile	71	67	67	2.16	10.8
Leu	73	61	73	3.42	17
Lys	72	69	67	3.03	15.1
Met	73	66	57	1.14	5.7
Phe	60	57	58	2.15	10.7
Thr	64	66	59	2.14	10.7
Trp	86	65	65	0.59	2.9
Val	74	66	68	2.48	12.4

1) Van Amburgh et al. [31] calculated from combined efficiencies for maintenance [77] and lactation [78].

2) Higgs and Van Amburgh [79]. Commercial ver. 7 of CNCPS is currently not available.

3) Amino acids, g/d relative to dietary metabolizable energy intake, Mcal/d.

4) Proportion of individual AA relative to total essential AA.

AA, amino acid; NASEM, National Academies of Sciences, Engineering, and Medicine; CNCPS, Cornell Net Carbohydrate and Protein System.

**Table 3:** Predicted animal variables, dietary nutrient composition, nutrient balance, and AA supply of diets formulated for dairy cows using NASEM [17] and CNCPS [31] models

Item	NASEM [17]		CNCPS (ver. 6.5.5) [31]	

	Basal	Basal+AA	Basal	Basal+AA
Predicted animal variables				
Energy allowable milk (kg/d)^1)^	47.6	47.9	47.0	47.2
MP allowable milk (kg/d)	36.5	37.6	45.0	45.6
Nutrient allowable milk (kg/d)	39.5	40.1	-	-
Predicted milk true protein (kg/d)	1.29	1.34	1.42	1.44
Predicted milk fat (kg/d)	1.42	1.44	1.60	1.59
Dietary nutrient composition (% DM)				
CP	15.4	15.7	15.5	15.6
ADF	21.7	21.6	21.0	20.9
NDF	33.2	33.1	30.6	30.5
Starch	25.6	25.6	26.3	26.2
Nutrient balance				
Energy (Mcal/d)^2)^	2.05	2.26	2.43	2.72
MP (g/d)	−422	−367	−0.40	32.4
Metabolizable amino acid supply (g/d)				
Arg	139	139	209	209
His	59	70	86	97
Ile	148	149	161	161
Leu	220	220	242	242
Lys	187	202	215	226
Met	54	65	69	79
Phe	141	142	158	159
Thr	131	131	152	152
Trp	32	32	45	45
Val	155	155	178	178

Diets formulated for a multiparous Holstein cow producing 45 kg/d milk yield, 4.2% milk fat, 3.4% milk true protein, 650 kg of body weight, at 3.0 body condition score and 150 days-in-milk, with 28 kg/d dry matter intake.

1) Net energy for lactation (NEL) allowable milk for NASEM [17] and metabolizable energy (ME) allowable milk for CNCPS.

2) NEL and ME for NASEM [17] and CNCPS [31], respectively.

AA, amino acid; NASEM, National Academies of Sciences, Engineering, and Medicine; CNCPS, Cornell Net Carbohydrate and Protein System; MP, metabolizable protein; CP, crude protein; ADF, acid detergent fiber; NDF, neutral detergent fiber.

## Data Availability

Upon reasonable request, the datasets of this study can be available from the corresponding author.
